# Domestic Quarter Horse (*Equus caballus*) Milk Macronutrient Composition Analyses Within Micro Quantities from Two Different Geographical Locations

**DOI:** 10.3390/ani15060882

**Published:** 2025-03-19

**Authors:** Jenna C. P. Wagner, Mark Edwards, Shweta Trivedi, Larry J. Minter, Kimberly Ange-van Heugten

**Affiliations:** 1 Department of Animal Science, College of Agriculture and Life Sciences, North Carolina State University, 120 W Broughton Dr, Raleigh, NC 27607, USA; jcpastel@ncsu.edu (J.C.P.W.); strived@ncsu.edu (S.T.); kdange@ncsu.edu (K.A.-v.H.); 2 Department of Animal Science, College of Agriculture, Food and Enviromental Science, California Polytechnic State University, 1 Grand Ave, San Luis Obispo, CA 93407, USA; msedward@calpoly.edu; 3 Animal Health Section, North Carolina Zoo, 4401 Zoo Parkway, Asheboro, NC 27205, USA

**Keywords:** lactation, mares, methods, nutrition

## Abstract

Microquantity laboratory procedures (<5 mL) are often used to analyze milk from non-bovid or less common species due to limited available samples and small sample sizes, unlike the large quantities of milk needed for macroquantity (~20 mL) assays standard in the commercial dairy industry. To better understand microquantity milk laboratory analyses, quarter horse (*Equus caballus*) milk from North Carolina State University (females = 4, n = 43) and California Polytechnic State University (females = 4, n = 42) equine centers was compared to investigate differences in macronutrient composition from 4 to 130 days after parturition. All mares were healthy, but consumed different diets. Major milk macronutrients were measured at the Smithsonian National Zoo and Conservation Biology Institute using a microquantity analysis of ash, crude protein (CP), dry matter (DM), crude fat, sugar, and gross energy. Of the six measured nutrients, only CP and crude fat differed (*p* < 0.05) by location, while sugar and crude fat had differences among individual mares. Californian mares had greater fat concentration means on a dry matter basis (DMB) than the North Carolinian mares (ANCOVA, *p* = 0.003). North Carolinian mares had greater CP concentration means than Californian mares. These findings indicate that dietary differences and/or environmental factors may play a role in CP and crude fat milk macronutrient composition within horse breeds. However, despite the differences noted, the microquantity analyses for nutritional means for all eight mares were within macroquantity horses ranges available in the previous literature. The results indicate that micro versus macroassays are comparable and supports clinical and scientific research on milk from species where large sample quantities are difficult to obtain.

## Simple Summary

Horses are valued companion, work, and agriculture animals. Investigations into horse milk’s nutritional composition are limited. In addition, most analyses of horse milk are performed with technologies that are standardized for bovine animals. This study assesses horse milk macronutritional composition between two locations on the opposite sides of the United States’ coasts using microquantity laboratory procedures.

## 1. Introduction

The horse (*Equus caballus*) was domesticated as early as 7000 BCE [1], and, since then, over 300 breeds [2] have been utilized globally for transportation [3], recreation [4], and food industries [5]. Research into the nutritional composition of horse milk has been conducted for three budding industries: (1) human consumption, (2) foal milk replacers for horse rearing, and (3) horse meat production [6]. Mare milk has become an increasingly recognized ingredient in health beverages for human consumption, leading to an increase in mare milk production [7]. Certain factors have helped spur this trend, including the production of cheese from mares’ milk [8] and the potential reduced allergic reactions humans may have to horse milk relative to traditional cow’s milk [9]. Mares’ milk has also been researched for foal milk replacers in case of orphanage, sickness, or general need of supplementation [10]. Equine milk formulas are commercially available for over-the-counter purchase and attempt to closely replicate horse milk nutritionally throughout the different phases of lactation [11]. Mare milk composition can vary over time, but can also vary depending on individual females and geographic location [10].

The analysis of mare’s milk is conducted using a variety of methods, with the most common being conducted by large-scale milk analysis companies. These laboratories analyze milk samples effectively, but require a minimum of 20 mL of milk for each sample [12]. In addition, most milk testing facilities are calibrated by cow milk standards, reflecting the dominance of cow milk in the industry [13]. This means that there are not readily available microquantity (<5 mL) analysis tools that are not influenced by cow standards.

When researchers have investigated the macronutrient composition of non-cattle milks, particularly from threatened species, the sample quantities are often small (<5 mL). This is due to the collection opportunities being restricted by species access, milk being vital for the neonate, and the inability to train and/or obtain willing samples from dams [14]. At the Smithsonian National Zoo and Conservation Biology Institute (SZCBI) (Washington, DC, USA), most of the archived milk samples were collected opportunistically [15]. Compared to a dairy farm, where approximately 8 gallons of milk [16] can be retrieved from one female cow a day using machine pumping, the act of milking a non-domestic or non-bovid species presents many more limitations. In addition to a lower sample quantity, there is also a concern with analysis that the laboratory standard curve range for macronutrient measurements encapsulates bovine milks. With this, standard curves cannot encompass the natural variation in macronutrient compositions present in the milks from other species that may contain macronutrient percentages outside of the bovine standard curve. Although there are various methods to analyze milk macronutrients with micro- and macroquantities, most agricultural laboratories use spectroscopy analysis, which requires macroquantities due to ease and cost [17,18,19]. There are established standards of cow milk nutritional composition that have inherently made cow milk the default for milk method testing.

Horse milk is an excellent model milk to assess the consistencies in milk macronutrient concentrations that could be impacted by bovine standards since it comes from a domesticated species and is easily available, despite being from a different phylogenetic group. The goal of this study is to (1) assess if common milk macronutrient composition analyses used for microquantity analyses are variable compared to larger labs using macroquantities, and (2) to determine if there is significant variation in horse macronutrient composition depending on individual females and geographical location (North Carolina (NC) vs. California (CA)). Therefore, the overall aim of this study is to pursue milk macronutrient composition using microquantity analyses from domesticated species as a model for other species in the future.

## 2. Materials and Methods

The study protocol was approved by North Carolina State University Animal Care and Use Committee (Approval number, 22-133; Approval date, 4 February 2022).

Quarter horse (*Equus caballus*) milk samples were acquired from eight single-parity healthy mares that did not twin. Four mares (NC1, NC2, NC3, and NC4) (age = 5–14; Body Condition Score (BCS) = 6–6.5) from the first geographic location were sampled at the North Carolina State University equine research unit in Raleigh, NC, USA. The mares were managed and cared for by the equine unit staff and volunteers. The mares were bred for educational and research purposes. They were not part of any treatment group. The mare’s diets ranged from receiving 2.72–5.54 kg of Purina Impact All Stages 12:6 Pellet (Purina, Arden Hills, MN, USA) per day. They also received approximately 2.72 kg of alfalfa (*Medicago sativa*) per day and had access to free-choice oat (*Avena sativa*) hay. The mares were housed in covered barns and were provided 24/7 access to pasture.

The four mares (CA1, CA2, CA3, and CA4) from the second geographic location were sampled at the California Polytechnic State University equine center, San Luis Obispo, CA, USA. These mares were managed and cared for by the equine unit staff and volunteers, bred for educational and research purposes, and were not part of any treatment group. The horses received 2 flakes (2.50 kg per flake) of alfalfa hay, 1000 g of proprietary in-house formulated grain mix (San Luis Obispo, CA, USA), and 66 g of Platinum Performance Equine (Platinum Performance, Buellton, CA, USA) feed in the morning, followed by 1 flake (2.04 kg per flake) of Bermudagrass (Cynodon dactylon) at midday, and then they were given 2 flakes of alfalfa and 1000 g of proprietary in-house formulated grain mix (San Luis Obispo, CA, USA) in the evening. These horses were housed in covered barns. The Bermudagrass pasture access was limited to 50% access a week before foaling; for 3–5 days post-parturition, the mares were kept in stalls, followed by 7 days of Bermudagrass pasture access for 50% of the days, then returning to 24/7 access to Bermudagrass pasture after 10–12 days post-parturition.

Before each milk collection, the teat area was cleaned with fresh water and thoroughly dried. Milk ejection was manually stimulated by applying pressure to the area surrounding the teat and squeezing firmly towards the end of the teat [20]. The milk was collected in clean Thermo Fisher Scientific (Waltham, MA, USA) 2 mL cryovials. Milk samples were taken from the mares every day up from 3 to 7 days after parturition. After day 7, the milk samples were taken every 3 days. Four weeks after parturition, milk sampling was conducted every seven days. This continued until approximately 130 days post-parturition. Milk samples specifically from NC were milked primarily in the afternoon; that information was not collected from CA. Mares foaled between February 2022 and April 2022, with the final milk collection taking place in August 2022.

Milk samples were frozen at approximately −28 °C until the end of the collection period and then shipped overnight to the Smithsonian Zoo and Conservation Biology Institute (SZCBI) with ice packs in insulated packaging. Milk macronutritional composition analyses were completed for dry matter (DM), crude fat, crude protein (CP), total sugar, gross energy (GE), and ash at the SZCBI Nutrition Laboratory (Washington, DC, USA) following the lab’s standard protocols with duplicates [17]. Crude protein was determined by multiplying the total nitrogen by the conversion factor 6.38 [21]. Total nitrogen was measured using an elemental analyzer (Model 2400; Perkin Elmer, Waltham, MA, USA). Fat content was measured with a modified microfat Rose-Gottlieb procedure [17]. Total sugars were measured using a phenol–sulphuric acid colorimetric procedure [16,22] and using lactose monohydrate standards and read at 490 nm on a microplate reader (Model ELX808; BioTek, Winooski, VT, USA). Ash was determined by placing dried milk in a muffle furnace at 550 °C for 8 h. Dry matter was both measured and calculated throughout sample analysis when drying the milks while subsampling for CP analysis and ash at 100 °C in a forced-air-drying oven. A GE formula verified against GE values measured by bomb calorimetry for milk from several species [23] was utilized for this study. The GE (kcal/g milk) was calculated using the following formula: GE = (9.11 kcal/g × % fat + 5.86 kcal/g × % CP + 3.95 kcal/g × % sugar)/100.

Macronutrient concentrations were assessed for GE, DM, CP, sugar, fat, and ash between 3 and 130 days post-parturition. The general concentrations of macronutrients were assessed on a milk basis, dry matter basis (DMB), and gross energy basis (GEB). Analysis was performed using R statistical software version 4.4.3 (The R Foundation, RStudio, Boston, MA, USA), and statistical results of means, medians, and ranges were found for individual mares and housing locations.

An analysis of covariance (ANCOVA) was conducted among individuals and between locations for each of the assayed macronutrients with a fixed factor of time. A post hoc Tukey’s honest significant difference test was conducted if the proceeding ANCOVA results were significant. Values were considered significant if the *p* value < 0.05. Multilinear regression models and Pearson correlations were determined to assess the relationships between macronutrient compositions over time.

## 3. Results

There was a total of eight individual horses, with at least ten samples analyzed between 4 and 130 days post-parturition (DPP) (Table 1). When comparing individual mares, there were 28 unique combination comparisons made among individuals. When comparing locations, there were an even number of individuals (four) for each group, representing two locations: California (CA) and North Carolina (NC). All foals were healthy, and there was no abnormal nursing behaviors reported.

When comparing milk dry matter percentage (DM%) among individual females (ANCOVA, *p* = 0.10) and between locations (ANCOVA, *p* = 0.13), there was no significant difference (Table 2). The change in DM% over time (DPP) was insignificant, but decreased (linear regression, R2 = 0.18, Pearson coefficient = −0.46). The average DM% was 11.09%.

There were no differences in GE DMB among individuals (ANCOVA, *p* = 0.17) nor location (ANCOVA, *p* = 0.059) (Table 2). There was no significant correlation between GE and days post-parturition (linear regression, R2 = 0.16, Pearson coefficient = −0.43). The average GE was 50.89 kcal/g.

The range for ash concentrations was between 0.63 and 7.02% DMB, with the average =3.69% DMB (0.40% of total milk). Ash concentrations were relatively constant over time on a DMB (linear regression, R2 = 0.19, Pearson coefficient = −0.62). There were no differences in ash concentrations (DMB) based on location (ANCOVA, *p* = 0.20) (Table 2) nor individuals (ANCOVA, *p* = 0.12).

**Figure 1:**
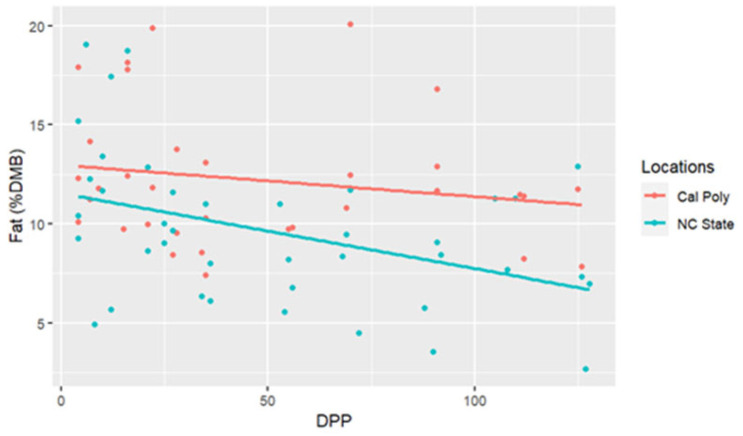
Percent fat concentrations (dry matter basis, DMB) of California Polytechnic State University (Cal Poly) and North Carolina State University (NC State) mares’ milk from 4 to 130 days post-parturition (DPP) with associated regression lines (Cal Poly = 4, NC State = 4) (n = 85 milk samples, ANCOVA, *p* < 0.05).

**Figure 2:**
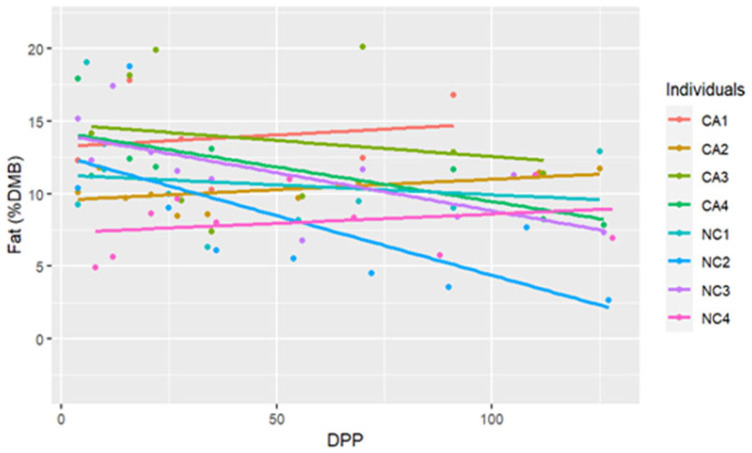
The trendlines and values of milk percent fat concentrations (dry matter basis, DMB) among eight individual horses from 4 to 130 days post-parturition (DPP) at two housing locations (California Polytechnic State University (CA) = 4 and North Carolina State University (NC) = 4) (n = 85 milk samples, ANCOVA, *p* < 0.05).

The range for fat concentrations was between 2.65% and 20.09% DMB, with the average = 10.61% DMB and 1.18% on a total milk basis. Fat concentrations did not have a significant change over time on a DMB (linear regression, R2 = 0.12, Pearson coefficient = −0.35). CA mares had significantly higher milk fat concentrations (DMB) than NC mares (ANCOVA, *p* = 0.003) (CA = 12.24%, NC State = 9.10%) (Table 2) (Figure 1). There were four differences in fat concentrations (DMB) among individual comparisons which were all interinstitutional: between NC2 < CA3 (P = 0.002), NC4 < CA3 (P = 0.01), NC2 < CA1 (P < 0.001), and NC4 < CA1 (P = 0.003) (Figure 2).

**Figure 3:**
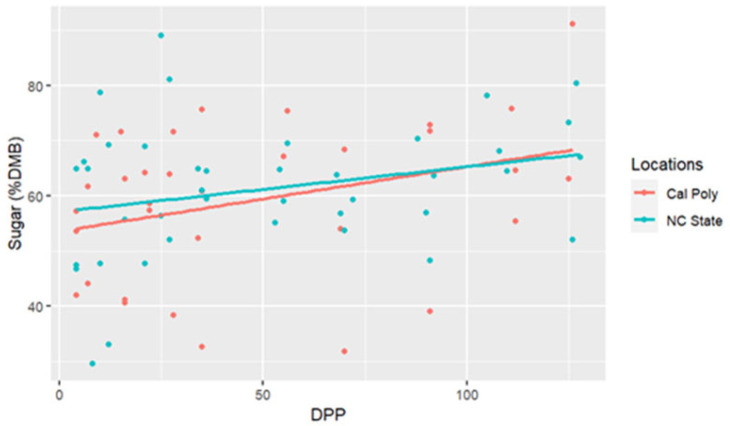
Percent sugar concentrations (dry matter basis, DMB) of California Polytechnic State University (Cal Poly) and North Carolina State University (NC State) mares’ milk from 4 to 130 days post-parturition (DPP) with associated regression lines (Cal Poly = 4, NC State = 4) (n = 85 milk samples, ANCOVA, *p* > 0.05).

**Figure 4:**
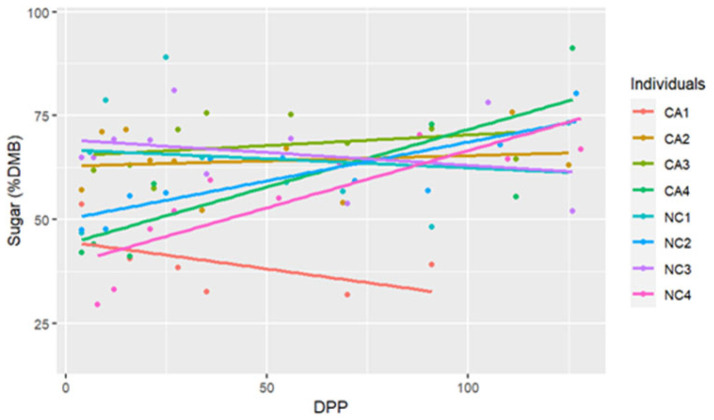
The trendlines and values of milk percent sugar concentrations (dry matter basis, DMB) among eight individual horses from 4 to 130 days post-parturition (DPP) at two housing locations (California Polytechnic State University (CA) = 4 and North Carolina State University (NC) = 4) (n = 85 milk samples, ANCOVA, *p* < 0.05).

The range for sugar concentrations was between 29.58% DMB and 91.20% DMB, with the average = 59.69% DMB (6.59% milk). Sugar concentrations were relatively constant over time on a DMB (linear regression, R2 = 0.075, Pearson coefficient = 0.29). There was no difference in milk sugar concentrations (DMB) based on location (ANCOVA, *p* = 0.11) (Table 2 and Figure 3). There were five differences in sugar concentrations (DMB) among individual comparisons; between CA1 < CA3 (*p* < 0.001), NC1 < NC2 (*p* = 0.02), CA1 < NC1 (*p* = 0.02), CA1 < CA2 (*p* = 0.001), and CA1 < NC3 (*p* < 0.001) (Figure 4).

**Figure 5:**
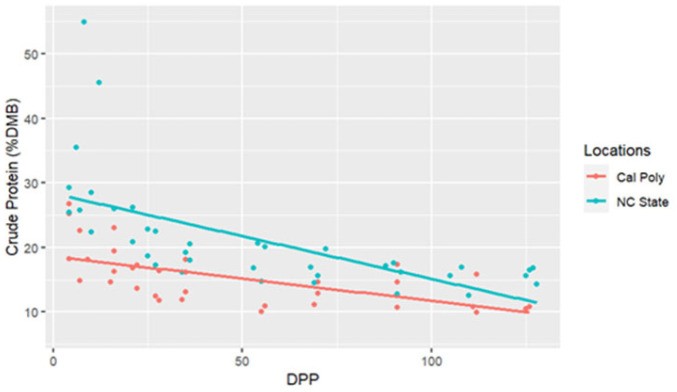
Percent crude protein concentrations (dry matter basis, DMB) of California Polytechnic State University (Cal Poly) and North Carolina State University (NC State) mares’ milk from 4 to 130 days post-parturition (DPP) with associated regression lines (Cal Poly = 4, NC State = 4) (n = 85 milk samples, ANCOVA, *p* < 0.05).

**Figure 6:**
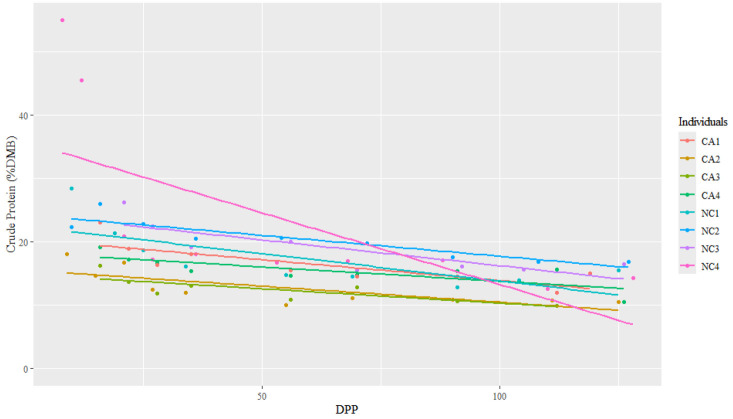
The trendlines and values of milk percent crude protein concentrations (dry matter basis, DMB) among eight individual horses from 4 to 130 days post-parturition (DPP) at two housing locations (California Polytechnic State University (CA) = 4 and North Carolina State University (NC) = 4) (n = 85 milk samples, ANCOVA, *p* > 0.05).

The range for CP concentrations was between 9.98 and 54.97% DMB, with the average = 19.88% DMB (2.24% of total milk). Crude protein concentrations were relatively constant over time on a DMB (linear regression, R2 = 0.14, Pearson coefficient = −0.38). California mares had significantly lower milk CP concentrations (DMB) than NC State mares (ANCOVA, *p* < 0.001) (CA = 15.68% DMB, NC = 20.48% DMB) (Table 2 and Figure 5); however, there were no differences in CP (DMB) among individuals (ANCOVA, *p* = 0.22) (Figure 6).

## 4. Discussion

Based on the results presented, microquantity measurement methods are effective for analyzing milk from mares and potentially related species. Milk assays for ash, fat, and CP were performed without traditional milk standardization protocols, which reduces dependency on machine calibrations [17]. In addition, these assays were performed in duplicates using less than 2 mL of milk. The general methods themselves also required minimal instrumentation and did not involve expensive equipment [23], making them accessible for laboratories or institutions that have limited resources. The microquantity measurements appeared to reliably execute mare milk macronutrient values that were within the estimated and normal ranges expected based on previous studies [24]. Furthermore, despite variations in diet and locations, the milk macronutrient content values obtained using microquantity methods were within in the ranges reported in previous studies [24,25,26,27]. The nutrient composition found was also consistent with the milk composition of a healthy mare and foal since there were no major developmental or medical concerns with the test subjects. Notably, colostrum (milk prior to day three in this study) was not included in this study.

The macronutrient composition among individual females were compared to determine if there was a major variance in milk macronutritional composition based on individuals. There were slight significant variations in sugar, fat, and DM concentrations; however, there were no individual trends or consistencies in the differences. In addition, the proportion of individuals that did have differences in macronutrient composition was lower than the proportion of individuals that did not have any differences (proportion different: DM = 3.6%, fat = 14.3%, sugar = 17.8%, CP = 0%, GE = 0%, ash = 0%). Although individual variation can influence milk macronutrient composition [21], it was not a significant factor based on this study. These findings are promising, suggesting that when making conclusions of overall species’ milk compositions, individual variation may be a minor factor.

Despite the differences in macronutrient composition among locations, the microanalysis values were within the ranges reported in the literature using macroanalysis assays [24,25,26,27]. Among the six macronutrients measured (DM%, sugar, CP, crude fat, ash, and GE), the only notable difference was that California mares had higher overall fat concentrations and lower overall CP concentrations compared to mares housed in North Carolina. There are several factors may influence milk macronutrient composition. In this study, we controlled for factors including the breed of horse [28], parity [29], number of offspring [30], seasonality [31], and days post-parturition [31]. However, in this study, we did not control the mare BCS [32], diets [33], age [34], time of collection [35] (including time of last suckling [36]), number of foals feeding [36], and total milk yield [37,38].

The cause of these differences in milk nutrition composition may be due to a difference in diet composition [32]. A prior study successfully manipulated mare milk fat and protein concentrations using diet changes including fat and fiber dietary supplementation and increased forage diet compared to concentrated pellets [27]. This suggests that diet changes can significantly impact mares’ milk macronutrient composition. Both locations provided similar quantities of alfalfa hay and extensive pasture access. However, feed intake trials were not conducted at either location; therefore, pasture grazing was estimated as 2.8% of body weight, as per the National Research Council (NRC) determination of a healthy grazing intake [39]. In the general diet assessment, pasture was assumed to be classified as “good” status. The two facilities provided a manufactured pelleted diet that differed between them in guaranteed analyses, and NC offered a larger quantity of pelleted feed. California also provided Platinum Performance Equine (a fatty acid supplement) (Platinum Performance, Buellton, CA, USA), where no equivalent additional feed supplement was provided for NC mares. After the diet software analysis, including pasture (used estimated values provided by software), hay (used estimated values provided by software), pellets, and supplements, the NC diets had a greater percentage of fat (CA = 3.7%; NC = 8.5%) and protein (CA = 24.7%; NC = 34.5%) on a dry matter basis than the CA diet. However, using a diet nutritional analysis, CA had a higher sugar content (Zoo Diet NaviGator Database Software Program (Version 2.4)) in their diets compared to NC diets (CA = 60.2%; NC = 46.7%). The increase in protein content in the milk from the NC mares is likely the result of an increased concentration of protein formulated into the NC State mare diets. However, the NC diet had a higher fat percentage despite the CA mares’ diet having a fatty acid supplement.

Mare milk fat composition may have been impacted by factors other than diet, including the time of milk collection [35], frequency of nursing [36], and overall milk yield [37,38]. In one study, mares’ milk was assayed within a 24 h period to determine the variation in fat and protein content, as well as milk yield throughout a day [40]. It was observed that fat content in the mares’ milk during the day was higher than that at night and that there was a reduction in fat content in the early morning [40]. The relationship between the fat content and time of day was also correlated with milk yield and fullness of the udder, where the higher the milk yield, the lower the fat content in mare’s milk [40]. However, NC mares were milked in the afternoon and last nursing was unknown, so this could not explain the lower milk fat content compared to the California mares. Another factor that could have contributed to the differences in the fat content between the locations is the method of milk collection. Since the foals were nursing ad libitum, controlling for milk yield and udder fullness was not feasible. The lower fat content in the NC mare’s milk may be attributed to the fact that residual fraction milk was not collected by emptying the udder [41]. In summary, several variables within this study could have influenced the differences in milk fat content between NC and CA mares, warranting future investigation.

This study faced limitations due to the small number of females sampled; however, this low sample size allowed for better control over boarding conditions. In addition, the comparison of microquantity analysis was only compared to macroquantity values in the literature. For future research, it would be advantageous to conduct a direct assessment using micro- and macroquantity analyses. We conclude that, although diet or location can alter the macronutritional composition of mare’s milk, such changes remain within the normal range for the species [24,25,26,27]. This finding is particularly relevant for studies analyzing small milk sample sizes from non-bovid species, where diet and location may vary. The ability to analyze milk in low quantities is particularly beneficial for future analyses of milk of endangered animals.

Furthermore, the reduced need for specialized equipment enhances accessibility for conducting assays in conservation institutions that may be more remote or have limited resources. This study supports the methodology and the accuracy of milk microassays with non-bovine milks.

## 5. Conclusions

There is promising evidence to support the future use of microquantity analysis to analyze mare milks. There were differences between locations in protein and fat content in the milk that could be explained by differences in animal diet and milk yield. Despite these differences, this methodology reproduced results that reflect the milk’s overall expected macro-nutritional value, based on previous values from the literature.

## Figures and Tables

**Table 1:** Milk samples analyzed for each individual mare represented over days post-parturition (DPP) timeline. The average number of milk samples per mare = 11 (total n = 85).

Individuals	DPP	n
CA1	4–119	11
CA2	4–125	11
CA3	4–112	10
CA4	4–126	11
NC1	4–125	11
NC2	4–127	10
NC3	4–126	11
NC4	4–128	10 ^1^

^1^ CA = mares from California Polytechnic State University, NC = mares from North Carolina State University.

**Table 2:** The total averages (%) of milk macronutrients on a milk (fresh) and dry matter basis (DMB) for all eight mares and at each institution (North Carolina State University (NC) and California Polytechnic State University (CA)) over 130 days post-parturition.

	Dry Matter	Sugar		Protein *		Fat *		Ash		Gross Energy
	%	Fresh	DMB	Fresh	DMB	Fresh	DMB	Fresh	DMB	Kcal/g
Average	11.09	6.63	60.36	2.35	20.65	1.20	10.77	0.41	3.69	50.89
NC	11.27	6.85	61.35	2.90	25.01	1.08	9.58	0.45	3.96	53.90
CA	10.86	6.36	59.16	1.68	15.37	1.34	12.20	0.37	3.36	47.26

* There is a significant difference between NC and CA mares (ANCOVA, *p* < 0.05).

## Data Availability

The raw data supporting the conclusions of this article will be made available by the authors on request.

## Abbreviations

The following abbreviations are used in this manuscript:

NC	North Carolina
CA	California
DMB	dry matter basis
GE	gross energy
